# Radioligand therapy (RLT) as neoadjuvant treatment for inoperable pancreatic neuroendocrine tumors: a literature review

**DOI:** 10.1007/s12020-022-03170-0

**Published:** 2022-08-26

**Authors:** Luca Urso, Alberto Nieri, Ilaria Rambaldi, Angelo Castello, Licia Uccelli, Corrado Cittanti, Stefano Panareo, Irene Gagliardi, Maria Rosaria Ambrosio, Maria Chiara Zatelli, Mirco Bartolomei

**Affiliations:** 1 Nuclear Medicine Unit, Department of Oncology and Specialist Medicines, University Hospital of Ferrara, Ferrara, Italy; 2 Translational Medicine Department, Ferrara University, Via L. Borsari, 46, 44121 Ferrara, Italy; 3 Department of Nuclear Medicine, Fondazione IRCCS Ca’ Granda, Ospedale Maggiore Policlinico, Milan, Italy; 4 Nuclear Medicine Unit, Oncology and Haematology Department, University Hospital of Modena, Modena, Italy; 5 Section of Endocrinology, Geriatric and Internal Medicine, Department of Medical Sciences, University of Ferrara, Ferrara, Italy

**Keywords:** Radioligand Therapy, Peptide Receptor Radionuclide Therapy, ^90^Y, ^177^Lu, Pancreatic Surgery, Neuroendocrine Tumors, Neoadjuvant Therapy

## Abstract

In the last 10 years, several literature reports supported radioligand therapy (RLT) in neoadjuvant settings for pancreatic neuroendocrine tumors (PanNETs). Indeed, primary tumor shrinkage has been frequently reported following RLT in unresectable or borderline resectable PanNETs. Moreover, RLT-induced intratumoral modifications facilitate surgery, both on primary tumor and metastasis, having a great impact on progression free survival (PFS), overall survival (OS) and quality of life (QoL). However, prospective controlled investigations are necessary to confirm preliminary data and to define the best RLT scheme and the ideal patient that, in a multidisciplinary approach, should be referred to neoadjuvant RLT.

## Introduction

Pancreatic neuroendocrine tumors (PanNETs) account for ~10% of all neuroendocrine tumors (NETs). Approximately 70% of these neoplasms are non-functioning and frequently present as relatively indolent and slow-growing masses. Therefore, diagnosis is frequently delayed, occurring when the disease has already spread to lymph nodes, liver, or bone [1–3].

PanNETs are currently classified according to the 2017 WHO classification. Grading has a strong impact on both clinical behavior and prognosis of patients affected by PanNETs, being a crucial parameter to take into consideration in the global therapeutic strategy [4].

Currently, surgery is potentially the only curative treatment for PanNETs [5]. Conversely, chemotherapy and molecular targeted agents are indicated in patients with advanced, recurrent or metastatic tumors not suitable for surgery (unresectable or borderline resectable), particularly in higher grade forms, while the role of external beam radiotherapy is limited [5–7].

Well-differentiated PanNETs usually overexpress somatostatin receptors (SSTRs), representing an ideal model for a theranostic approach in Nuclear Medicine. At present, procedures are well established for radiolabelled somatostatin analogs (SSA) with single-photon emission computerized tomography (SPECT) or positron emission tomography (PET) agents, such as Indium-111 (^111^I) or Gallium-68 (^68^Ga). Likewise, in the settings of radioligand therapy (RLT), SSA can bind high energy beta emitters, such as Yttrium-90 (^90^Y) or Lutetium-177 (^177^Lu) [8, 9].

Since 2017, after the encouraging results from the NETTER-1 phase III clinical trial, RLT has been established as an effective and safe therapy for advanced, metastatic or inoperable gastro-entero pancreatic (GEP) NETs [10–12]. RLT is usually proposed as a delayed treatment, when other lines of therapy have already failed [13]. Nevertheless, before NETTER-1 several studies reported a greater efficacy of RLT if offered at a relatively earlier stage of disease, when the tumor burden is lower and patients have a better performance status [14, 15]. Therefore, based on literature evidences, RLT has been proposed as a neoadjuvant treatment, in order to obtain a tumor size decrease or downstaging, allowing a surgical approach and increasing the likelihood of a radical resection [3, 16–20].

The present mini-review explores the emerging role of RLT as a potential neoadjuvant treatment for PanNETs.

## Methods

We performed a literature search using MEDLINE (PubMed database) on April 30^th^ 2022 to identify articles in English on neoadjuvant RLT. We used Keywords chosen according to Medical Subject Heading (MeSH) terms “neoadjuvant” OR “preoperative” AND “radioligand therapy” OR “Peptide Receptor Radionuclide Therapy”. We included original articles and case reports. Editorials, Letters, and abstracts from conference proceedings were excluded. The full articles of selected studies were examined, and additional searches of their reference lists performed to identify other potentially eligible articles. Moreover, research of active ongoing trials was performed on clinicaltrials.gov.

## Results

We found 11 papers including 148 patients as reported in Table 1. Literature evidence is heterogeneous considering sample size, patient characteristics, RLT schemes and outcome assessment. Our analysis and considerations were focused on the following issues: a) tumor response: primary shrinkage, downstaging and downgrading; b) histological and tumor microenvironment modifications; c) influence on tumor burden; d) safety and reproducibility of neoadjuvant RLT.

**Table 1:** Literature review of neoadjuvant RLT in PanNETs

Study	Year	Patients treated	Radionuclide used	Outcome	Toxicity
Kaemmerer et al. [21]	2009	1	^90^Y-DOTATOC (2 cycles)	CR after surgery	Only mild transient hematologic toxicity
Stoeltzing et al. [34]	2010	1	^90^Y-DOTATOC (2 cycles)	CR after 1 year from surgery	Not reported
Ezzidin et al. [22]	2012	1	^177^Lu-DOTATATE (3 cycles)	Inoperable PAN-NET became eligible for surgery achieving a complete local remission for 22 months	Minor transient leukopenia (Grade 1)
Barber et al. [28]	2012	5 (4)	^177^Lu-DOTATATE (1–4 cycles)	3 scintigraphic PR; 4 radiological PR and 1SD. 1 patient affected by PanNET became eligible for surgery with a reduction in Ki67 from 10–15% to 5%.	Not reported
Van Vliet et al. [23]	2015	29	^177^Lu-DOTATATE (3–4 cycles)	9/29 patients became amenable for surgery and had an increase in PFS vs. control group (69 vs. 49 months)	No patients showed delayed toxicity
Partelli et al. [16]	2018	23	^177^Lu/90Y-DOTATATE (20/3 pt.)	• No pancreatic fistula development in RLT patients. • Longer PFS in RLT patients with R0 resection.	Only 1 patient had hematologic toxicity and stopped RLT
Da Silva et al. [24]	2018	1	^177^Lu-DOTATATE	Patient became eligible for surgery and was tumor-free after 3-year follow up	No RLT-induced toxicity reported
Zanata et al. [25]	2021	1	^90^Y-DOTATOC	Patient became eligible for surgery and was tumor-free after 108 months follow up. Disease downstaging from stage IV to stage I.	Not reported
Parghane et al. [26]	2021	57 (32)	^177^Lu-DOTATATE	6/32 PanNETs became resectable after RLT. Primary tumors and liver metastasis decreased in size.	Not reported
Opalinska et al. [27]	2021	9 (6)	^177^Lu/90Y-DOTATATE	Median primary tumor size decreased by 1.6 cm after RLT, with no differences between Pan-NETs and other GEP-NETs. 4/9 patients became resectable after RLT and in 2 a radical surgery was achieved. Liver metastases became undetectable in 2 patients.	No myelotoxicity or nephrotoxicity found
Minczeles et al. [29]	2022	49	^177^Lu-DOTATATE	Patients with neoadjuvant RLT followed by surgery had a longer PFS and OS than those who performed only RLT. A downstaging was found in the 38% of patients with baseline tumor-vessel involvement	47% of patients had transient hematologic toxicity. G3 liver toxicity was found in 1 patient

Treated patients: the number within brackets indicates the number or PanNETS among the cohort of patients treated with RLT.

*CR* complete response, *PanNET* pancreatic neuroendocrine tumors, *PFS* progression free survival, *PR* partial response, *RLT* radioligand therapy, *SD* stable disease.

Moreover, consulting clinicaltrials.gov we found 1 active prospective multicentre trial concerning neoadjuvant RLT for PanNETs.

### Tumor response: primary shrinkage, downstaging and downgrading

**Fig. 1:**
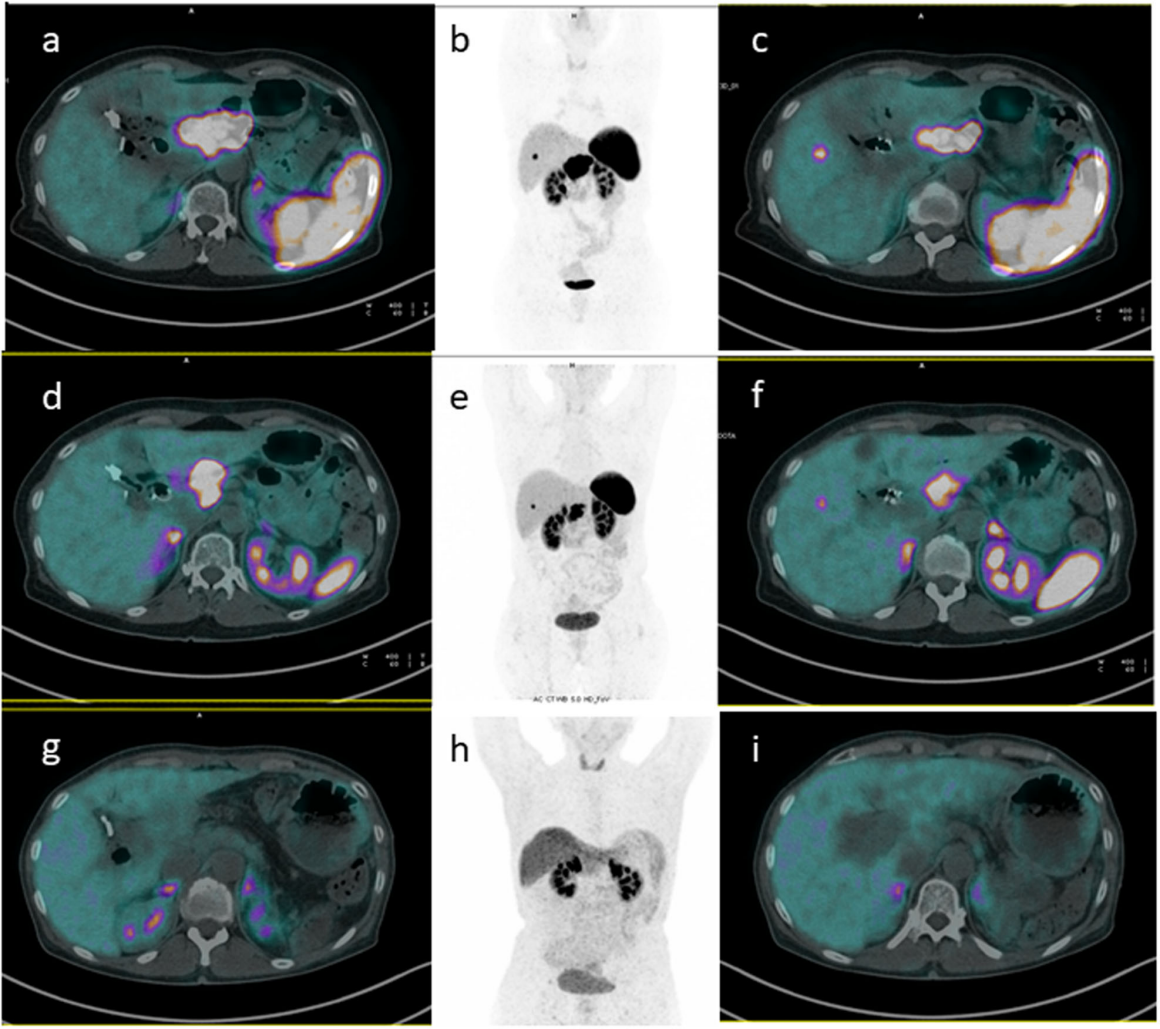
A successful case of neoadjuvant RLT. **a–c** Baseline [^68^Ga]-DOTATOC PET/CT of a patient with a primary NET mass of the pancreatic head (SUVmax 72) and a liver metastasis (SUVmax 24). **d–f** [^68^Ga]-DOTATOC PET/CT examination after V cycles of ^90^Y- and ^177^Lu RLT, showed a receptor density and dimensional reduction in pancreatic mass (SUVmax 56) and hepatic lesion (SUVmax 17). The patient then underwent second-line surgery. **g–i** [^68^Ga]-DOTATOC PET/CT scan one year after surgery well demonstrated a complete macroscopic response, with no pathological uptake

Overall 148 patients with locally advanced PanNETs undergoing neoadjuvant RLT are described in literature. Seventy-two became eligible for surgery [16, 21–29]. Of note, we found 2 cases of extremely large primary tumors who became eligible for surgery following a significant tumor shrinkage and reduction in vascular involvement [24, 25]. Indeed, RLT acts as a cytoreductive agent in well-differentiated NETs, leading to a volumetric reduction of target lesions (Fig. 1). The possibility of performing curative or debulking surgery, in this subset of patients, is associated with an improvement in survival rates and quality of life (QoL), even in advanced or metastatic disease [25]. Moreover, Partelli et al. [16] reported that, among patients with a curative resection (i.e. R0), those treated with neoadjuvant RLT showed a significantly longer progression free survival (PFS) than those who performed surgery up-front. Similarly, Minczeles et al. [29] reported that patients with neoadjuvant RLT followed by surgery had a longer PFS and OS than those who performed RLT only.

The same authors also demonstrated a downstaging in 38% of patients with baseline tumor-vessel involvement.

Grading is a key parameter to assess NETs aggressiveness and, consequently, prognosis [4]. A few papers reported downgrading after RLT [21, 25, 28], mostly from G2 to G1. The reduction in Ki-67 index may be due to the greater radiosensitivity of tumor cells characterized by a higher proliferating cell population [16, 30]. Therefore, we can speculate that neoadjuvant RLT in PanNETs might act by preserving low-grade cell clones compared to the high-grade ones, which are more radiosensitive and selectively killed. The resulting global downgrading obtained could potentially have a significant impact on long surviving patients’ outcomes, both in terms of PFS, OS and QoL [25]. Nevertheless, further studies with larger number of cases are required to validate this hypothesis.

### Histological and tumor microenvironment modifications

As reported by Schiavo Lena et al. [31], neoadjuvant RLT can induce intratumoral microenvironment modifications and changes in stroma/cellularity ratio, inducing a progressive scarring process and fibrotic involution. Of note, stromal percentage was higher in patients treated with neoadjuvant RLT in comparison with those who underwent surgery up-front, particularly in patients in which a remarkable reduction in tumor diameter was achieved. Such tissue modifications, together with tumor size decrease can simplify surgery, reducing both operative time and major complications, particularly pancreatic fistula [16]. As a consequence, patients treated with neoadjuvant PRRT could benefit of a faster post-operative recovery, with a concomitant cost reduction for the Healthcare System.

### Influence on tumor burden

RLT is a systemic therapy targeting tumor cells expressing SSTRs. In patients with oligometastatic NETs, neoadjuvant RLT could therefore potentially target both primary tumor and metastases. Particularly, liver tumor burden is well known as a main prognostic factor in NETs [12, 30, 32]. Recently, the North American Neuroendocrine Tumor Society suggested several potential advantages of neoadjuvant RLT in reducing liver tumor burden in metastatic PanNETs [33]. In this subset of patients RLT might allow subsequent locoregional hepatic therapies with a potential curative outcome and a consequent increase in PFS, OS and time to recurrence [26, 33]. This recommendation is supported by the positive results obtained with neoadjuvant RLT in reducing liver disease burden, as previously reported [26–28, 34]. Moreover, RLT-induced biological modifications can cause liver metastases necrotic involution, avoiding the need of a subsequent therapeutic approach [31]. Theoretically, a similar outcome could be pursued with neoadjuvant chemotherapy, as described by Squires et al. [35], who reported promising results combining neoadjuvant capecitabine and temozolomide in PanNET. Moreover, recent evidence suggests that capecitabine may act as a radiosensitizer in synergy with RLT, increasing the efficacy of this approach in aggressive or radioresistant NETs [36]. Therefore, these results suggest that a combined approach with RLT and chemotherapy in neoadjuvant settings could be effective. Indeed, Barber et al. [28] achieved functional/radiological and symptomatic response over 12–42 month combining neoadjuvant RLT and chemotherapy (5-Fluorouracil). However, further prospective studies, comparing and combining neoadjuvant RLT and neoadjuvant chemotherapy approaches in patients with systemic disease at diagnosis are needed to explore this therapeutic option. A multidisciplinary approach is always preferable for selecting the best treatment and the correct timing.

### Safety and reproducibility of neoadjuvant RLT

Among available studies, only one reported that a single patient was withdraw from neoadjuvant RLT before completing the treatment, due to severe hematologic toxicity [16]. Mild and transient hematologic toxicity was reported in other studies [21, 23, 24, 27, 29], in line with the most updated clinical and dosimetric evidence [37–39].

The available results derive from studies with heterogeneous neoadjuvant RLT schemes. Indeed, different isotopes have been employed: ^90^Y in 3 studies [21, 25, 34], ^177^Lu in 6 [22–24, 26, 28, 29] and both radioisotopes in 2 [16, 27], always as mono-therapy. It should be noted that, overall, most of the patients (141/148) were treated with [^177^Lu]-labeled radiopeptides. On the other hand, very different schemes were employed concerning cycles number and injected activity. Therefore, a better standardization of neoadjuvant RLT is desirable and should be investigated in future trials. Although RLT is currently mainly performed with [^177^Lu]-based radiopharmaceuticals, [^90^Y]-radiolabeled peptides might still play an important role. Indeed, the greater tissue penetrance of the beta radiations emitted by ^90^Y, compared to ^177^Lu, enhances the cross-fire effect that damages even those cellular clones with lower SSTR expression and potentially more undifferentiated. This feature, combined with the higher linear energy transfer (LET), candidates [^90^Y]-radiopeptides as potentially more effective in the treatment of large and biologically aggressive lesions [8, 40]. On the other hand, [^177^Lu]-based RLT has proven to be safer than ^90^Y. ^177^Lu could be more effective on small lesions, also in relation to its longer half-life, allowing a prolonged dose delivery [41]. Baum et al. [8] reported greater benefits in terms of objective response and survival in a large series of patients affected by NET (including PanNETs) who received combination treatments with [^177^Lu]-DOTATOC and [^90^Y]-DOTATOC, but not in neoadjuvant settings. A dual-isotope approach could allow a better risk-benefit ratio in neoadjuvant setting in PanNETs, representing a challenge for planning future multicentre randomized trials in this subset of patients.

### Ongoing trials

On the basis of available evidence, a phase II single-arm multicentric trial on neoadjuvant RLT with [^177^Lu]-DOTATATE, followed by surgery for PanNETs, (NEOLUPANET, EUDRACT 20019-002196-34 – Clinical Trials Identifier NCT04385992) is ongoing [42]. The main purposes of the study are to evaluate safety and efficacy of neoadjuvant RLT followed by surgery in resectable PanNETs at high-risk of recurrence. The secondary endpoints are to investigate the rate of objective radiological response to RLT according to modified RECIST criteria (mRECIST) and patients QoL. The results of this study are not available, yet.

Further ongoing trials proposing neoadjuvant RLT for PanNETS or for NETs of different primary origin are not available at present. Likewise, other trials aiming to compare the efficacy of different RLT schemes in this subset of patients are lacking.

### Limits of the study

The present mini-review summarizes the available data in the literature. However, most of the studies on neoadjuvant RLT are single case reports or small sample size studies [16, 21–29, 34]. Therefore, the quality of the evidence is quite low. On the other hand, prospective randomized clinical trials are difficult to plan for these patients.

## Conclusions

RLT could represent a safe and effective neoadjuvant therapy in unresectable or border line resectable PanNETs. Further prospective controlled investigations are necessary to confirm these preliminary data and to define the ideal therapeutic scheme. In addition, the multidisciplinary approach is preferable to identify patients who could benefit from neoadjuvant RLT to become eligible for second-line surgery.
